# Machine learning strategy to improve impact strength for PP/cellulose composites via selection of biomass fillers

**DOI:** 10.1080/14686996.2024.2351356

**Published:** 2024-05-08

**Authors:** Koyuru Nakayama, Keita Sakakibara

**Affiliations:** Research Institute for Sustainable Chemistry, National Institute of Advanced Industrial Science and Technology (AIST), Hiroshima, Japan

**Keywords:** Lignocellulose, cellulose nanofiber, polymer composite, impact energy, machine learning, infrared spectroscopy

## Abstract

Lignocellulosic materials have inherent complexities and natural nanoarchitectures, such as various chemical constituents in wood cell walls, structural factors such as fillers, surface properties, and variations in production. Recently, the development of lignocellulosic filler-reinforced polymer composites has attracted increasing attention due to their potential in various industries, which are recognized for environmental sustainability and impressive mechanical properties. The growing demand for these composites comes with increased complexity regarding their specifications. Conventional trial-and-error methods to achieve desired properties are time-intensive and costly, posing challenges to efficient production. Addressing these issues, our research employs a data-driven approach to streamline the development of lignocellulosic composites. In this study, we developed a machine learning (ML)-assisted prediction model for the impact energy of the lignocellulosic filler-reinforced polypropylene (PP) composites. Firstly, we focused on the influence of natural supramolecular structures in biomass fillers, where the Fourier transform infrared spectra and the specific surface area are used, on the mechanical properties of the PP composites. Subsequently, the effectiveness of the ML model was verified by selecting and preparing promising composites. This model demonstrated sufficient accuracy for predicting the impact energy of the PP composites. In essence, this approach streamlines selecting wood species, saving valuable time.

## IMPACT STATEMENT

This paper introduces a data-driven method to efficiently design lignocellulosic polymer composites with high-impact energy, optimizing components and surface areas using infrared spectroscopic data.

## GRAPHICAL ABSTRACT

**Figure:**
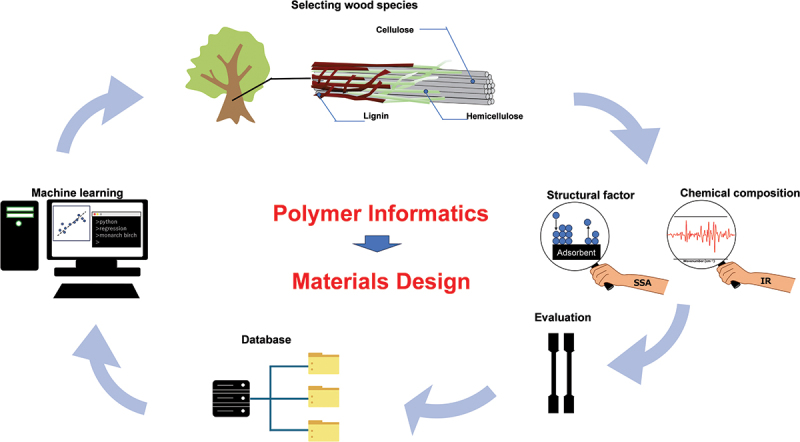

## 1. Introduction

**Figure 1.**
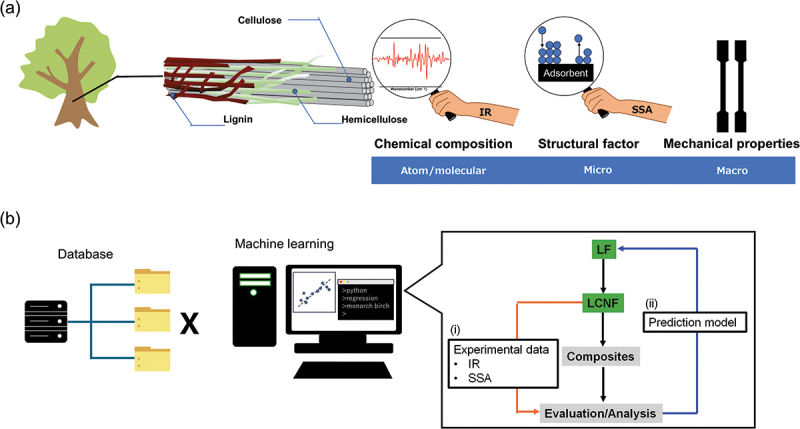
(a) Natural nanoarchitecture in LFs and LCNFs with relationship between mechanical properties. (b) Data-driven strategy in this study.

A growing interest has been in utilizing lignocellulose fillers (LFs) derived from wood biomass as thermoplastic reinforcement [1,2]. These natural fillers offer numerous advantages, including abundance, renewability, low specific gravity, high specific strength and stiffness, and cost-effectiveness, enabling lightweight construction designs and reducing petroleum resources. LFs have been converted to nanofibrillated fillers, which are called lignocellulose nanofibers (LCNFs), via mechanical fibrillation processes, such as wet disk-milling, gaining significant attention as superior reinforcements in olefin-based polymer nanocomposites compared to non-/less-fibrillated lignocellulosics [3–5]. Since these LFs and LCNFs have inherent complexities and natural nanoarchitectures, such as a variety of chemical constituents in wood cell walls (cellulose, hemicellulose, lignin, and others), various structural factors such as fillers (fiber width/length, specific surface area (SSA), crystallinity index (CI), etc.), surface properties (wettability, reactivity, etc.), and variation in production (wood species, provenance, production year, etc.), comprehensive utilization of such natural fillers for polymer composites is not straightforward, requiring laborious trial-and-error optimization process to achieve good dispersion in thermoplastics (polyethylene, polypropylene (PP), etc.), and thus high mechanical properties (modulus, strength, impact energy, etc.) (Figure 1(a)). Furthermore, further steps need to be optimized in producing polymer composites, such as the base material selection, filler pretreatment, and composite processes [6].

Several studies have been conducted to address this challenge to improve the mechanical properties of LFs/LCNFs – reinforced polymer composites [7]. Among various mechanical properties, enhancing impact properties is a crucial issue directly linked to the durability and safety of the products [8]. Since composites are inherently complex because of their numerous parameters, one strategy is understanding how specific major parameters could affect impact property. For example, variations in constituent components, such as the cellulose/lignin/hemicellulose ratio in biomass fillers, affect the mechanical properties of these polymer composites materials, in which a complexity extends to differences among wood species [9–14]. A comparative review of lignocellulose composites has highlighted superior impact strength [15]. The SSA, fiber shape, and filler dispersibility within the base material significantly affect the mechanical properties of the composites [16]. Pavithran et al. emphasized the contribution of larger angles of spiral cellulose microfibrils in cell walls to a higher fracture toughness [17]. Mueller et al. showed that higher fiber fineness in composites has been linked to enhanced impact performance [18]. In a broader context, the influence of external temperature and water on the impact properties of LFs/LCNFs-reinforced thermoplastics has been thoroughly reviewed [19]. Despite valuable insights into lignocellulose composites, the nanoscale implications of the impact energy remain relatively unexplored and warrant further research. This is because the impact property should be related not only to one parameter but also to several parameters that are connected to each other dependently. Thus, notable discrepancies exist between the theoretical predictions and the experimental results for composites. The complexity arises from various factors, including filler size [20], interactions [21], chemical properties [22–24], and microscopic spatial arrangement (dispersion, aggregation, percolation), making them difficult to characterize [25,26]. This complexity raises questions regarding the accuracy and reliability of predictive models.

Recently, machine learning (ML) has emerged as a powerful tool for addressing the computational challenges enabled by the advancements in big data science [27,28]. ML methods have shown potential for addressing complex problems, particularly inorganic materials with disordered microstructures, such as glasses and alloys [29–31]. These techniques excel in processing high-dimensional input vectors and can effectively uncover the intricate relationships, potentially contributing to figure out the impact properties of LFs-/LCNFs-reinforced polymer composites related to several parameters. Nevertheless, the application of ML to polymer materials, particularly in polymer composites, has limitations [32]. A primary hindrance is the time-consuming nature of data acquisition in this domain. Moreover, even when past data are available, their utility is restricted by their inadequacy because of substantial process variability inherent in material manufacturing. Consequently, exploring the potential of ML in the context of polymer composites is necessary to overcome these challenges and unlock new opportunities for the rapid and efficient development of polymer composites.

In this study, we aimed to develop a novel and versatile ML strategy to improve mechanical properties, especially impact property, for LFs-/LCNFs-reinforced PP composites, which is highly demanding as an alternative for conventional talc-/glass fiber-reinforced PP composites used in automobiles, daily goods, and others. Figure 1(b) shows the scheme of this study. Firstly, in Figure 1(b) (step i), we focused on the influence of natural supramolecular structures in each wood species related to chemical compositional characteristics and fiber size properties of LFs and LCNFs, where Fourier transform infrared (FTIR) and SSA data are effectively used as spectral and filler information for the developing ML, on the mechanical properties of the PP composites based on principal component analysis (PCA). Subsequently, in step (ii) in Figure 1(b), the effectiveness of the ML model was verified by selecting and preparing promising LFs and LCNFs-PP composites. We emphasize that this study’s original contribution lies in employing IR spectra to predict the mechanical properties of plastic/wood composites in the process simply and successfully, which is a challenging task since there are various factors affecting the properties of raw materials and final products during the process. Moreover, it is possible to incorporate information about the material manufacturing differences using SSA, reflecting different fibrillation processes. Although the combination of IR with ML and PCA have been utilized to identify wood species [33] or to evaluate the enzymatic efficiency for saccharification [34], this study demonstrates another challenging linkage between raw materials and composites. This study offers a novel approach to constructing a physical property prediction model based on woody raw material information for polymer composites.

## 2. Experimental section

### 2.1. Basic data for prediction models

The data for the characterization of 14 kinds of lignocellulosic fibrillated fillers obtained by different mechanical processes, including dry pulverization (Dry-P) and subsequent wet disk milling for four and ten passes (Wet-DM-4, Wet-DM-10) (Table S1), and the mechanical properties, including tensile, flexural, and Izod impact properties of PP/lignocellulosic fibrillated fillers composites (Table S2), are based on the results obtained from the Development of Technologies for Manufacturing Processes of Chemicals Derived from Inedible Plants Project, commissioned by the New Energy and Industrial Technology Development Organization (NEDO), Japan (No. JPNP13006), which were collected before this study. Table 1 lists the 14 raw materials of cellulose used in this study. Figure S1 shows the morphology of selected typical LCNFs using scanning electron microscope (SEM).

**Table 1.** Cellulosic raw materials used for prediction models.

No.	Name	Provenance	Species	Age^a^	Abbreviation
1	Japanese cedar (*Cryptomeria japonica*)	Ibaraki, Japan	Softwood	J	JC-I-J
2				M	JC-I-M
3		Toyama, Japan		J	JC-T-J
4				M	JC-T-M
5		Kumamoto, Japan		M	JC-K-M
6	Chinese fir (*Cunninghamia lanceolata*)	Kochi, Japan		J	CF-J
7	Japanese larch (*Larix kaempferi*)	Nagano, Japan		J	JL-J
8				M	JL-M
9	Sakhalin fir (*Abies sachalinensis*)	Hokkaido, Japan		J	SF-J
10				M	SF-M
11	White birch (*Betula platyphylla*)	Hokkaido, Japan	Hardwood	J	WB-J
12				M	WB-M
13	*Eucalyptus nitens*	New Zealand		J	EN-J
14	Moso bamboo (*Phyllostachys edulis*)	Ibaraki, Japan	Glass	–	MB

^a^J: juvenile wood (part: 1–15 years); M: mature wood (−16 years).

### 2.2. FTIR spectroscopic data for prediction models

Attenuated total reflection (ATR) FTIR analyses of the 14 types of lignocellulosic fibrillated fillers after carrying out Dry-P, Wet-DM-4, and Wet-DM-10 in the dry state using a Frontier instrument (PerkinElmer Inc., U.S.A.). All spectra were obtained by accumulation of 32 scans in the 4000–600 cm^−1^ range. The resolution was 4 cm^−1^. All the IR spectra obtained for each sample (14 types × 3 processes = 42 samples) were transformed as follows.

#### 2.2.1. Standard normal variate (SNV) transformation

SNV is used not only to eliminate the effects of uneven particle distribution, surface scattering, and varying particle sizes on the spectrum but also to remove the influence of optical path reflection on the diffuse reflectance spectra. SNV operates by subtracting the mean value of the entire spectrum to remove a constant offset term and then dividing it by the standard deviation of the entire spectrum, scaling all spectra to the same level. The SNV transformation was applied to the spectral data and is expressed as

(1) $$X_{i,\mathrm{SNV}}=\frac{X_{i,k}-X_{i}}{\sqrt{\frac{\sum_{k=1}^{m}{\left(X_{i,k}-X_{i}\right)}^{2}}{m-1}}},$$

where $X_{i,\mathrm{SNV}}$ is the SNV-transformed value of the i-th data point in a specific spectrum$,X_{i,k}$ is the original intensity of the i-th data point in the k-th spectrum, $X_{i}$ is the mean intensity value of the i-th data point across all *m* spectra$,\left(\sum_{k=1}^{m}{\left(X_{i,k}-X_{i}\right)}^{2}\right)$ calculates the sum of the squared differences between each $X_{i,k}$ and the mean $X_{i}$ for all *m* spectra, where *m* denotes the total number of spectra, and $\left(\sqrt{\frac{\sum_{k=1}^{m}{\left(X_{i,k}-X_{i}\right)}^{2}}{m-1}}\;\right)$ is the standard deviation of the i-th data point across all *m* spectra.

### 2.3. PCA

PCA is crucial for studying trends, groupings, and outliers in large bilinear data structures. It is possible to determine the main variations in a multidimensional dataset by creating principal components (PC), which are new linear combinations of the fundamental latent structure of the original raw data in PCA. This enables the recognition of the characteristics and emphasizes the correlation with the physicochemical properties of the samples. This technique helps interpret ATR-FTIR spectra, in which the diversity and complexity of bands appear based on the source of the samples. The spectral data used in the PCA were based on the ATR-IR data obtained using the Savitzky – Golay (SG) filter for smoothing and secondary differentiation. The PCA and SG filters were computed using Python 3.10.

### 2.4. Autocorrelation matrix

Correlation analysis is a statistical method used to assess the strength and direction of the relationships between two variables. This relationship is quantified using the correlation coefficient, varying within a range of −1 to + 1. A correlation coefficient of ± 1 indicates a perfect relationship between the variables, with the sign ’+’ or ‘−’ denoting the direction of this relationship. The value of 0 indicates the absence of any relationship between the variables. The Pearson’s correlation coefficient is used under the assumption that the variables under consideration are normally distributed, making it the most applied measure of correlation in statistical analyses, and expressed as the following equation:

(2) $$r_{\mathrm{xy}}=\frac{n\sum x_{i}y_{i}-\sum x_{i}\sum y_{i}}{\sqrt{\left(n\sum x_{i}^{2}-{\left(\sum x_{i}\right)}^{2}\right)\left(n\sum y_{i}^{2}-{\left(\sum y_{i}\right)}^{2}\right)}},$$

where the Pearson correlation coefficient, denoted by $r_{\mathrm{xy}}$, quantifies the linear relationship between two variables x and y, n represents the number of observations, and $x_{i}$ and $y_{i}$ are the values of variables x and y for the i-th observation, respectively. Comprehensive computation of these coefficients was facilitated using Python 3.10, ensuring an exhaustive pairwise correlation analysis within the dataset.

### 2.5. Construction of prediction model by ML

Python 3.10 was used to construct the ML model for the Izod impact energy of the lignocellulosic fibrillated filler-reinforced PP composites based on experimental data (SSA in Table S1, Izod impact energy in Table S2, and PC1 and PC2 data from the PCA-analyzed second deviated IR spectra (Figure S2), and that for the tensile breaking strain (SSA and CI in Table S1, tensile breaking strain in Table S2, and PC3 data from the PCA-analyzed second deviated IR spectra (Figure S2)).

#### 2.5.1. Multiple linear regression (MLR)

MLR is used to predict the linear relationship between a dependent variable and multiple independent variables. In MLR, the independent variables influence the dependent variable. Therefore, the independent variable can be used as a predictive factor when the relationship with the dependent variable is validated. Constant and regression coefficients were calculated for each variable to explain the relationship between independent and dependent variables. The general multiple regression equation is given by

(3) $$Y=\beta_{0}+\beta_{1}X_{1}+\cdots +\beta_{n}X_{n}+\varepsilon ,$$

where *Y* is the dependent variable, *β*_0_ is the constant, *β*_1_ to *β*_n_ are the regression coefficients, *X*_1_ to *X*_n_ are the independent variables, and ε is the error term. In the case of the Izod impact energy prediction model, SSA of the lignocellulosic fibrillated fillers listed in Table S1, PC1, and PC2 from IR were *X*_1_, *X*_2_, and *X*_3_, respectively, and the Izod impact energy listed in Table S2 for PP/lignocellulosic fibrillated fillers composites were *Y*. In the case of the tensile breaking strain prediction model, SSA and CI for the lignocellulosic fibrillated fillers listed in Table S1 and PC3 from the IR were *X*_1_, *X*_2_, and *X*_3_, and the tensile breaking strain listed in Table S2 for the PP/cellulosic fibrillated fillers was *Y*. The experimentally obtained data (14 types × 3 processes = 42 samples) were randomly divided into two groups: training data sets (70%; 29 data sets) and testing data sets (30%; 13 data sets) for predicting the Izod impact energy or tensile breaking strain.

In these analysis examples, they are given by

(3)-i $$Y_{\mathrm{Izod}\;\mathrm{impact}\;\mathrm{energy}}=\beta_{0}+\beta_{1}X_{\mathrm{SSA}}+\beta_{2}X_{\mathrm{PC}1\left(\mathrm{IR}\right)}+\beta_{3}X_{\mathrm{PC}2\left(\mathrm{IR}\right)}+\varepsilon$$

(3)-ii $$Y_{\mathrm{tensile}\;\mathrm{breaking}\;\mathrm{strain}}=\beta_{0}+\beta_{1}X_{\mathrm{SSA}}+\beta_{2}X_{\mathrm{CI}}\text{\textbackslash{}break}\;\;\;\;\;+\beta_{3}X_{\mathrm{PC}3\left(\mathrm{IR}\right)}+\varepsilon$$

### 2.6. Discovering promising composites by data-driven approach

Approximately 50 wood plates (Table S3) purchased from Takada Seizaijo (Fukuoka, Japan) with dimensions of 135 × 180 × 12 mm (i.e. width × length × thickness) were used to evaluate the robustness of the Izod energy prediction models. These wood plates were subjected to ATR-FTIR measurements, and the IR spectra were converted to second-derivative spectra via SNV transformation. Subsequently, the PCA data with the average SSA, grouped in softwood and hardwood with the same fibrillation condition, were applied to the Izod impact energy prediction model (Equation 3-i). Because of the inherent difficulty in accurately measuring the SSA for empirical demonstration, the average values from the dataset were used in the calculations. These included a SSA of 5 m^2^/g for all wood species with Dry-P, 20 m^2^/g for softwoods with Wet-DM-4, 12 m^2^/g for hardwoods with Wet-DM-4, 118 m^2^/g for softwoods with Wet-DM-10, and 46 m^2^/g for hardwoods with Wet-DM-10. For the sample S3, S24, and S43 for RUN1 (Table S3) and S9, S30, S33, and S34 (Table S3) for RUN2, the SSA values were determined by the BET (Brunauer – Emmett – Teller) method using a BELSORP-mini (MicrotracBEL Corp., Japan). The freeze-dried filler samples of approximately 150 mg were heated at 105°C under vacuum for at least 3 h to remove adsorbed species. Nitrogen adsorption/desorption data were collected, and the BET equation was used for calculating SSA.

In RUN1, six specimens from three tree species (two processes) were selected based on predicted values, S3, S24, and S43 (Table S3 and Table 2), and subjected to experiments as above. Subsequently, the additionally-obtained data including the results of Izod impact energy and SSA were added to the dataset of the ML prediction model for RUN2. Then, RUN2 involved subjecting around 45 specimens, by subtracting the three specimens from RUN 1’s validation tests from Table S3, to PCA and conducting multiple regressions similarly (Figure 5(c)). Then, four tree species (two processes) were selected based on predicted values, S9, S30, S33, and S34 (Table S3 and Table 2), and subjected to experiments as above. Subsequently, the additionally-obtained data including the results of Izod impact energy and SSA were added to the dataset of the another ML prediction model.

**Table 2.** Results of two-round practical evaluation (RUN1 and RUN2) showing SSA, predicted and measured Izod impact energy values for the ML-selected LCNFs (Wet-DM-4)/PP composites as the experimental demonstration using multiple regression model, eq. 3-i.

	Sample ID	LCNF raw materials	Process		SSA (m^2^/g)	Izod impact energy (kJ/m^2^)	
						Predicted	Measured
RUN1	S3	Ginkgo		Dry-P	8.21	1.20	1.57
				Wet-DM-4	14.7	1.20	2.18
	S24	Japanese chestnut		Dry-P	3.11	1.75	1.79
				Wet-DM-4	13.3	1.75	1.82
	S43	Yellow birch		Dry-P	7.82	1.78	1.81
				Wet-DM-4	12.0	1.81	1.84
RUN2	S9	Spruce		Dry-P	8.52	1.07	2.11
				Wet-DM-4	18.1	1.03	2.07
	S30	Monarch birch		Dry-P	7.82	1.84	2.43
				Wet-DM-4	16.9	1.86	2.39
	S33	Paulownia		Dry-P	8.45	2.01	2.20
				Wet-DM-4	42.3	2.04	2.31
	S34	Persimmon		Dry-P	8.21	1.50	2.22
				Wet-DM-4	14.7	1.52	2.20

### 2.7. Production of lignocellulosic fibrillated filler-reinforced PP composites

Isotactic PP (NOVATEC MA3, Japan Polypropylene Co., weight-average molecular weight: ca. 397,000; density: 0.900 g/cm^3^; melt flow rate (MFR): 11) and maleic acid-modified PP (MAPP, Kayabrid 006PP, Kayaku Akzo Chemical Co., Ltd., ca. 0.6 wt% MA; density: 0.950 g/cm^3^; MFR: 115) were used without purification. Fibrillation, compounding, injection molding, and Izod impact measurements were conducted similarly to the reported condition [35], described in detail in the SI.

### 2.8. Fractography using SEM images

The morphological characteristics of the fractured surfaces of composite samples that underwent an Izod impact test were observed using a field emission SEM (FE-SEM, S-4800, Hitachi High-Technologies Co., Ltd., Tokyo, Japan). An acceleration voltage of 1.0–3.0 kV was used. The samples were coated with around 3 nm thick layer of osmium using an osmium plasma coater (NEOC-AN, Meiwa Fosis Co., Ltd., Tokyo, Japan).

## 3. Results and discussion

### 3.1. Dataset

In the dataset (see 2.1 Basic data for prediction models), Japanese cedar (JC), Chinese fir (CF), Japanese larch (JL), Sakhalin fir (SF), white birch (WB), *Eucalyptus Nitens* (EN), and moso bamboo (MB) are listed as raw materials (Table 1). The raw materials varied in terms of their provenance and age, classified as either juvenile wood (abbreviated as ‘J’, part: 1–15 years) or mature wood (abbreviated as ‘M’, over 16 years). Each material is abbreviated using a combination of the species name, provenance, and age category. For instance, juvenile wood of the JC species sourced from Ibaraki is designated as ‘JC-I-J’. Table S1 lists the characterization obtained from pristine wood materials with each pretreatment, where three kinds of filler, such as the fibrillated LFs (100-μm pass wood flour obtained by dry fine pulverization) and LCNFs obtained by the wet-DM method for 4- and 10-times treatments, are used for the PP composites. LCNFs are manufactured by mechanical processing from LFs without pulping or chemical processing. Table S2 summarizes the various mechanical properties obtained from the composites, where the filler content is 5 wt% in LFs, LCNFs-reinforced PP composites. In general, the continued disintegration of fibers results in notable trends within their mechanical properties: an increase in Young’s modulus is observed; in flexural tests, a similar upward trend is noted, with both the modulus of elasticity and maximum strength showing increases. These mechanical changes are influenced by a range of factors: the inherent properties of the filler, its reactivity with the compatibilizer MAPP, and the level of dispersion, to name a few. However, the Izod impact energy displays less predictable behavior. This unpredictability is particularly pronounced when considering impact performance, making it challenging to develop a predictive model for material properties based solely on these data. Consequently, implementing ML techniques is viewed as an effective solution to address these complexities. Wood comprises three major molecules (i.e. cellulose, hemicellulose, and lignin) with different properties and specific FTIR absorptions [36].

**Figure 2.**
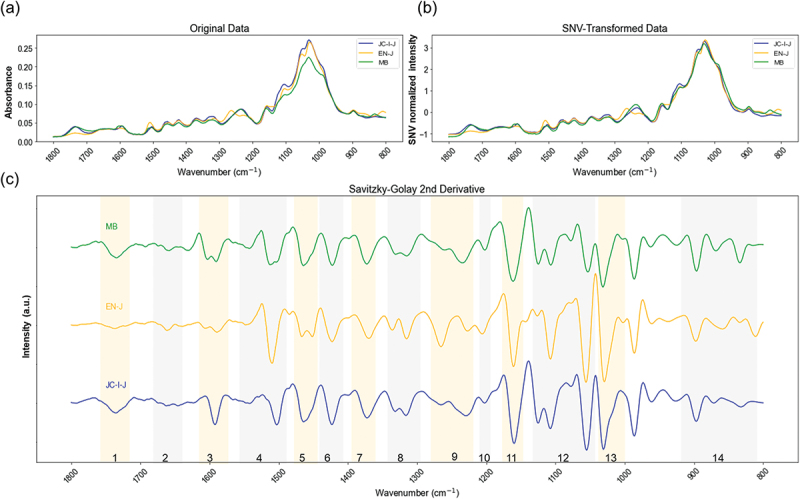
Spectrum analysis from (a) original, (b) SNV-transformed, and (c) SG-filtered second derivative spectra. Annotation 1–15 in Figure 2c (bottom) is referred to Table S4.

Figure 2 shows the FTIR spectra of typical plant biomasses from softwood (JC-I-J), hardwood (EN-J), and glass (MB). The major bands of the three main components are listed in Table S4. The spectra obtained in this study (Figure 2(a)) were subjected to SNV transformation as part of the experimental methodology to minimize measurement errors (Figure 2(b)). Evidently, the compositions of the respective source materials are highly similar, as indicated by their nearly identical IR spectra (Figure 2(b)). Therefore, the second derivative of the spectra was used as a band-narrowing/peak-sharpening method to identify hidden peaks (Figure 2(c)). We observed that even though the spectra in Figure 2(b) appear similar at first glance, differentiation becomes apparent upon conducting a second derivative analysis, particularly in the regions highlighted by the orange and gray bands, where odd numbers were highlighted in orange and even numbers in gray in reference to the band regions attributed in Table S4 for visual clarity. This finding is significant as cellulose and hemicellulose exhibit similar absorption bands, which tend to overlap as a broad single peak in the original data. However, the second derivative analysis allows for characterizing peak shapes and asymmetries, effectively distinguishing between these components. In the following section, we will show that ML made it possible to connect the different features of these second-deviation absorption bands with physical property data.

### 3.2. Data mining

#### 3.2.1. Correlation coefficient

**Figure 3.**
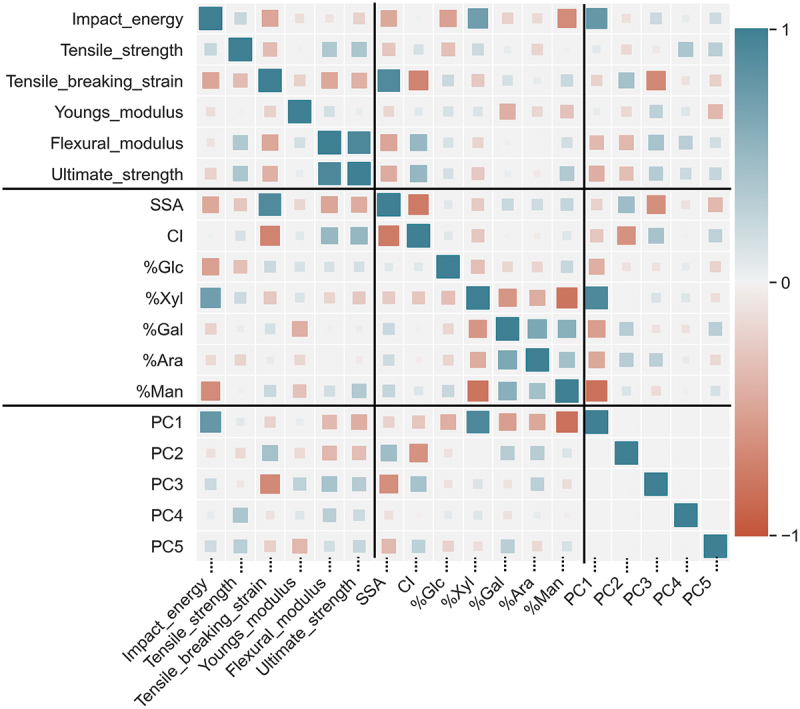
Heatmap of the correlation matrix generated by the Pearson correlation coefficient for mechanical properties, specific surface area (SSA), crystallinity index (CI), and sugar composition. The scale is set from − 1 (red) to 1 (blue), and the squares sizes express the number scales.

Data-driven approaches, such as statistical analysis and ML, are based on the correlations inherent in the dataset. Each composite dataset was evaluated using Pearson correlation coefficients to identify interesting correlation coefficients between pairs of properties (Table S1, S2), as shown in Figure 3. These coefficients included some that were expected and others that were newly discovered. The Pearson correlation coefficients in Figure 3 show the blue patches for positive correlations, red patches for negative correlations, and white patches for uncorrelated coefficients. The sizes of the patches reflect the magnitude of the correlation coefficient. Collinearity analysis showed no highly correlated properties except those obtained from the same measurements. As expected, the physical property parameters calculated from the same measurement showed large and small correlations, such as the tensile stress – tensile breaking strain, tensile breaking strain – Young’s modulus, and flexural modulus – ultimate strength. Parameters derived from similar experiments, such as Young’s modulus- flexural modulus and tensile strength- ultimate strength, also showed predictable correlations.

The high positive correlation between the SSA and tensile breaking strain is a fascinating insight. There are several papers that focus on the correlation in-between SSA and properties, for example, an increase in SSA results in stronger surface energy [37]. While this could imply easier aggregation effects, surface energy also influences polymer relaxation [21,38]. With careful preparation, cellulosic fillers could form strong interfacial strength with polar sites like MAPP, affecting dispersibility due to increased repulsion with PP. However, to the best of our knowledge, there were no specific studies focusing on correlation between the SSA and the strain of composites using CNFs, so the correlation between them remains unclear.

Furthermore, the amounts of sugar composition %xylose unit (%Xyl) and %mannose unit (%Man) differed between softwoods and hardwoods, exhibiting a correlation with mechanical properties, including impact energy. The %Xyl and impact energy show a highly positive correlation, whereas %Man is negatively correlated with Young’s modulus. These unexpected findings suggest significant differences between softwood and hardwood in the mechanical properties of the PP composites. It is noted that while some aspects regarding the constituent components and properties are described in the referenced paper [39], there are still unclear areas. Thus, this study lies in the ability to capture trends conveniently from the correlation coefficient heatmap generated in this analysis.

Consequently, it is desirable to predict the material properties based on the chemical composition of fillers. However, component analysis, such as sugar composition, is time-consuming and costly. The IR method discussed in Section 3.2.1 offers a more convenient alternative. Moreover, it provides more information than the sugar composition analysis. Therefore, we proceeded to investigate a method for examining correlations using IR data based on PCA.

It should be noted about the limitation of the relatively small sample size (42 specimens), which may introduce errors in the analysis. However, as stated by Tian et al., increasing data set size reduces errors in polymer material design, with optimal accuracy achieved with 30 to 40 samples in some cases [40]. To use the same facility and equipment, and accumulating high-precision data, we emphasize that it was possible to effectively utilize ML even with a small dataset. For future prospects, it is imperative to complement datasets to achieve more accurate predictions of material properties. One approach is to use artificial intelligence to conduct virtual experiments by generating data from techniques such as IR spectroscopy and SSA, thereby increasing the number of samples [40].

#### 3.2.2. PCA

**Figure 4.**
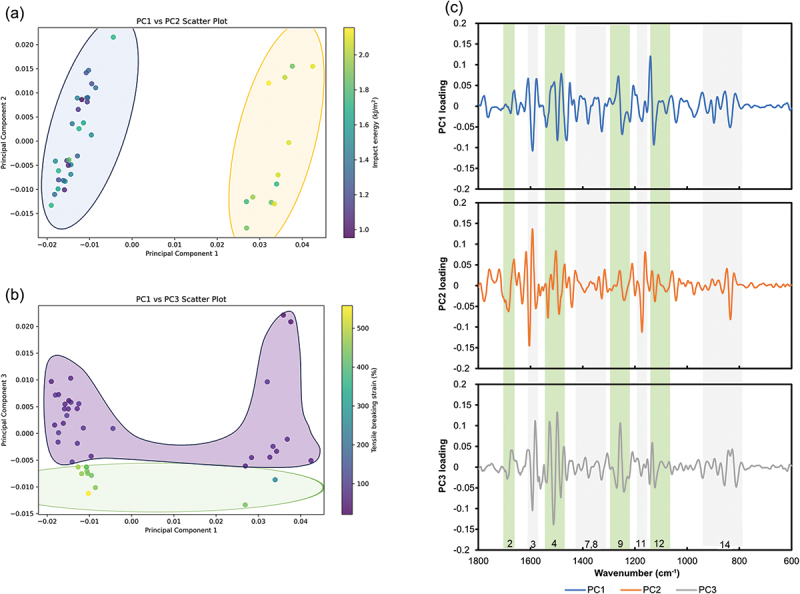
PCA plots for the 42 samples analyzed in this study. a) PCA score plot depicting PC1 versus PC2, b) PCA score plot depicting PC1 versus PC3, and c) a PC loading plot based on the ATR-FTIR spectra, which underwent preprocessing involving normalization and application of a Savitzky-Golay filter for second derivative transformation and smoothing. Shading in plots a, b) demonstrates the grouping based on impact energy and tensile breaking strain, respectively. It should be noted that the colored ellipses in these plots serve solely for illustrative purposes.

PCA is a dimensionality-reduction algorithm that projects the original space of predictors onto a lower-dimensional subspace. Linear regression on high-dimensional data (millions per sample) is inappropriate for standard regression due to high variability in goodness of fit, model overfitting on the training set, and poor prediction accuracy on the test set (resulting in a usable model for that material only). By sacrificing some finer details by performing PCA, the new set of features provides a more practical representation of the data. Standard linear regression is easy to implement but has significant limitations in terms of predictive power because of the linearity assumption. Figure 4 shows a score plot for PCA and a loading plot for ATR-IR for the 42 different wood species. The summarized results of PCA for IR spectroscopy are illustrated in Figure S2. In Figure 4(c), peaks correlating with PCs values were identified in regions labeled 2, 3, 4, 7, 8, 9, 11, 12, and 14 in the loading plot of the second derivative ATR-IR spectrum. Apparently, peaks correlating with lignin’s characteristic features were captured (regions 2, 3, 4). Furthermore, despite the structural similarities between cellulose and hemicellulose, the segregation of softwoods and hardwoods (including bamboo) along the PC1 direction, as illustrated in Figure 4(a), suggests a stronger reflection of the hemicellulose components.

The results of PCA for IR spectroscopy are incorporated into Figure 3 to reinforce these intriguing correlations. Notably, PC1 successfully segregated the groups, with softwoods, distinguished by %Man or %Xyl, in the negative direction and hardwoods in the positive direction. The hardwoods-derived LFs and LCNFs/PP composites exhibited relatively high impact energies when color-coded by impact energy values. Furthermore, examining the information from PC1 revealed correlations with other sugar compositions, suggesting that IR contains a wealth of information. These findings imply correlations between compositional components and material properties and the potential for obtaining information beyond sugar composition analysis using IR data.

### 3.3. Prediction model

**Figure 5.**
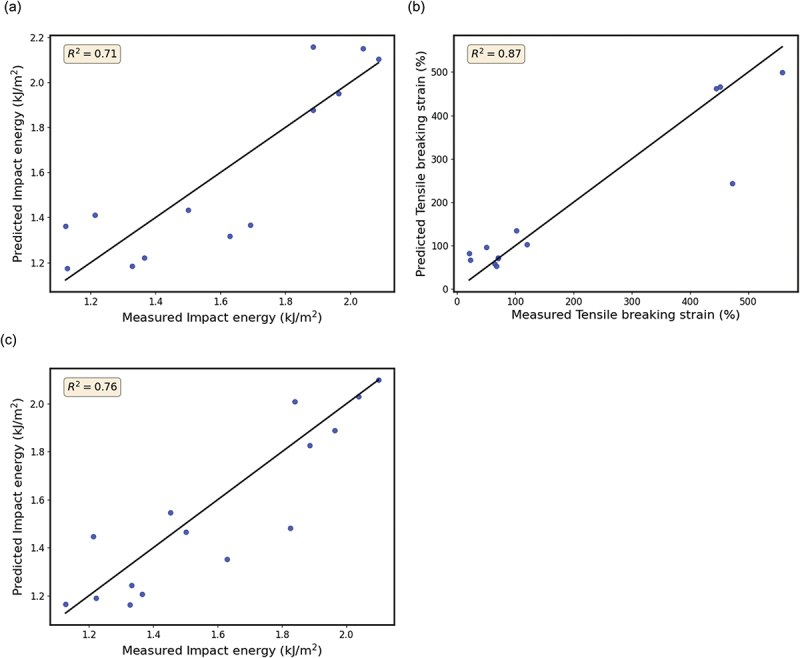
Relationship between the predictive and measured values from models for a) the impact energy (kJ/m^2^), and b) for the tensile breaking strain. c) Relationship between the predictive and measured values from models for the impact energy (kJ/m^2^), in which the results of practical evaluation from the RUN 1 were incorporated into the dataset for the prediction model: blue circles: test data; black line: prediction model.

We developed a model to predict the impact test results for lignocellulosic fibrillated fillers-reinforced PP composites. We utilized three independent variables, namely the SSA and the principal component scores (PC1 and PC2), obtained from the second derivative IR spectra. To ensure the impartiality of the test set selection process, we randomly divided the entire sample of 42 specimens into two subsets. The training data subset comprised 70% of the total sample, and the test data subset accounted for the remaining 30% (randomly selected, as shown in Table S2). We used the training data subset to calculate the constant coefficients β_i_ for the regression Equation (3) to minimize the total squared error. We focus on developing predictive models for Izod impact energy and tensile breaking strain, which hold significance in composites. These predictive models are shown in Figure 5. Initially, the multiple regression analysis for impact energy prediction yielded an R-squared (R^2^) value of approximately 0.70 in Figure 5(a). Figure 5(b) presents a regression model for predicting the tensile breaking strain of the composites developed using a similar methodology. The model exhibits a high R^2^ value of 0.87, indicating substantial predictive accuracy. However, it should be noted that this high level of accuracy may partially stem from the distinct clarity between good and poor experimental results, which tends to facilitate the model’s ability to select higher values predominantly from the extreme ends of the data range. Thus, the missing measurement data within the range of 150–450% may play a significant role in the regression. By incorporating additional data, particularly in the range of 200–400, there is a promising opportunity to develop a truly predictive model that is applicable for practical use. Our findings provide a foundational understanding for future research, paving the way for further investigations that can build on the intricate details uncovered in this study.

### 3.4. Practical evaluation

This study used the multiple regression model to predict the impact energies of previously unknown 45 wood species listed in Table S3. Every wood species was investigated by ATR-FTIR, and the prediction model was applied. Finally, some woody fillers were chosen for practical evaluation in PP composites (Table 2). For RUN1, Gingo (S3), Japanese chestnut (S24), and yellow birch (S43) were selected based on property predictions obtained from multiple regression analysis. Among these, S24 and S43 yielded measured values close to the predicted values.

The results of practical evaluation from the RUN1 were further incorporated into the dataset to construct the model for RUN2 (Figure 5(c)). For RUN2, Spruce (S9), monarch birch (S30), paulownia (S33), and persimmon (S34) were selected based on new property predictions obtained from multiple regression analysis. Monarch birch and persimmons have emerged as promising candidates. Notably, the performance of S30, with a predicted value of 1.84 and 1.86 compared to an actual measurement of 2.43 and 2.39 for Dry-P and Wet-DM-4, respectively, is significant. This represents a significant improvement, exceeding 190%, in contrast to the measured value of 1.26 for the neat PP. However, it is essential to acknowledge that the predicted values tended to be consistently higher overall. The Izod impact energy values for PP in the dataset and recently experimentally obtained data were 1.05 and 1.26, respectively, suggesting that the different lot for PP pellets could affect the impact test. The improvement in the R^2^ value, mainly achieved by using IR and SSA in RUN1, supported the utility of this information. While the primarily dataset mainly focused on softwoods, the dataset became considerably enriched through the practical evaluation (RUN1 and 2), comprising 12 softwood species, 9 hardwood species, and 1 glass species, totaling 56 specimens (42 specimens in Table S1 + 14 species in Table 2) across different processes. Although the cause of some discrepancy between predicted and measured impact energy values in Table 2 is unclear, it suggests the existence of properties beyond those assessed using IR and the SSA used in the property predictions. Identifying and incorporating these additional properties into future models is challenging for ongoing research.

**Figure 6.**
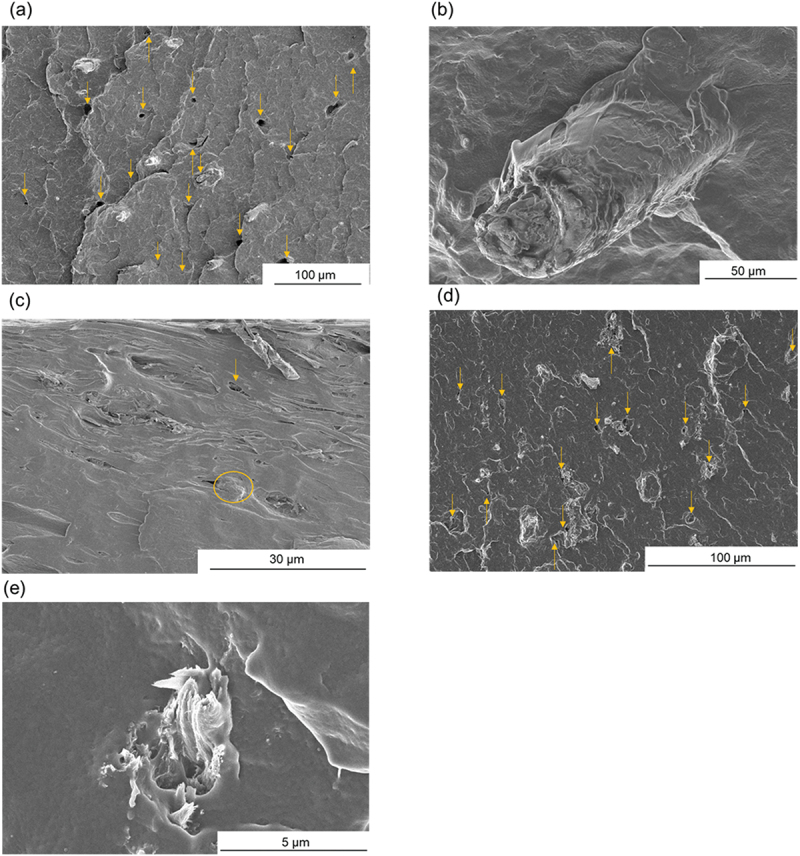
FE-SEM images for fractured surfaces of a, b) MB Wet-DM-4, c) JC-I-M dry-P, and d,e) Monarch birch Wet-DM-4 reinforced PP composites. The images b, e) are magnified views. The yellow arrows in Figures a), c), and e) indicate holes formed due to fibers detaching from the sample surface after the impact test. The yellow circle in Figure c) indicates the tip of a fiber in the PP matrix.

The fractography underwent an impact test using SEM images, as shown in Figure 6. In MB with Wet-DM-4/PP composites, which is in the category of higher impact energy composites, there was significant fiber exposure in the fractured surface, along with observable holes due to fiber pullout (Figure 6(a)). Moreover, the MB fibers exposed in the fracture were longer at their bases (Figure 6(b)). In contrast, the JC-I-M (Dry-P)/PP composite sample, which is in the category of lower impact energy composites, exhibited less protruded fibers and holes from the fracture surface, indicating good adhesion properties between the fillers and PP (Figure 6(c)). The circled area represents the tip of a fiber in Figure 6(c). It was thus inferred that fracture predominantly occurred within the matrix, particularly in areas with fewer adhesive interfaces at the fiber tips. In the case of Monarch Birch (S30)/PP composites, the number of fibers and holes observed on the fracture surface were somewhat noticeable (Figure 6(d)). The size and quantity of holes were greater than those in JC-I-M with Dry-P/PP composite. Upon examining magnified images, fiber breakage/tearing seems to be apparent in monarch birch-derived composite (Figure 6(e)), a phenomenon not often seen in MB Dry-P/W-DM-4, JCI-M Dry-P or JL-M W-DM-4 composite samples. This suggests that the fibers may have absorbed impact through tearing in monarch birch. The impact causes the fibers to detach moderately, thereby dispersing energy effectively. It is noteworthy that through ML, samples were efficiently extracted and analyzed, facilitating the acquisition of these important insights.

## 4. Conclusions

From the dataset for 42 wood samples/PP composites were used for establishing prediction models. The distinctive aspect of this study is the selection of descriptors using correlation coefficients, encompassing both IR spectroscopy and SSA data for lignocellulosic fibrillated fillers, integrated for regression analysis. In IR spectroscopy, second derivative spectra were employed to differentiate components like hemicellulose from cellulose despite their similar peaks. Furthermore, we enhanced the clarity of spectral features by applying PCA for dimension reduction. The explanatory variables for ML were refined using correlation coefficients. The prediction technique investigated a developed multiple regression analysis model constructed from SSA, PC1, and PC2 derived from IR spectra. This model demonstrated an R^2^ greater than 0.7, indicating sufficient accuracy for predicting impact energy. In essence, this approach streamlines selecting wood species, saving valuable time. While the test’s applicability varies based on its intended use, evaluating the physical properties of materials in controlled environments under extreme conditions benefits greatly from models encompassing a wide range of environmental conditions. Such models enable a more precise and comprehensive assessment.

In this study, we confirmed a remarkable enhancement in impact energy, reaching the highest levels observed. Specifically, compared to PP, monarch birch demonstrated an improvement of over 190% against neat PP, affirming the model’s efficacy. This consistency can be attributed to the strong correlation between the data and the formed structures. However, discrepancies were noted when the predicted values of the physical properties were compared with the actual measurements. It is hypothesized that this divergence was influenced by data not utilized in the current analysis, suggesting room for improvement.

## Supplementary Material

Supplemental Material
