# Supplementary material for: Machine learning strategy to improve impact strength for PP/cellulose composites via selection of biomass fillers
Source: Sci Technol Adv Mater. 2024 May 8;25(1):2351356. doi: 10.1080/14686996.2024.2351356 (PMC11138231; doi:10.1080/14686996.2024.2351356)
Supplement: Supplemental Material [file TSTA_A_2351356_SM6486.pdf]

Supporting information (SI) for:

Machine learning strategy to improve impact strength for PP/cellulose composites via selection of biomass fillers

Koyuru Nakayama* and Keita Sakakibara*
Research Institute for Sustainable Chemistry, National Institute of Advanced Industrial Science and Technology (AIST), 3-11-32 Kagamiyama, Higashi-Hiroshima, Hiroshima 739-0046, Japan.
*Corresponding Authors
Koyuru Nakayama, nakayama-koyuru@aist.go.jp
Keita Sakakibara, sakakibara.keita@aist.go.jp

This PDF file includes:

1. Supporting Experimental Procedures
2. Supporting Tables
3. Supporting Figures
4. Supporting References

1. Supporting Experimental Procedures

1.1 Data for impact prediction models

The data for the characterization of 14 types of lignocellulosic fibrillated fillers with different mechanical preparations, including dry pulverization and subsequent wet disk milling for four and ten passes (Table S1), and the tensile and Izod impact properties of PP/lignocellulosic fibrillated filler composites (Table S2) were based on the results obtained from the Development of Technologies for Manufacturing Processes of Chemicals Derived from Inedible Plants Project, commissioned by the New Energy and Industrial Technology Development Organization (NEDO), Japan (No. JPNP13006).

1.2 Demonstration for higher impact energy composites

Fibrillation of lignocellulosic fillers

Wood flour (particle size <0.2 mm) was used as the raw material for preparation of LCNFs. Six types of wood samples (Table 3) were selected using the prediction model, eq. 3-(i), processed using a cutter mill (MKCM-3, Masuko Sangyo Co., Ltd., Japan), and an air jet mill (MKCL8-15J, Masuko Sangyo, Japan). The wood flour was soaked in distilled water (3 wt. %), and passed through a disk mill (MKCA6-3, Masuko Sangyo, Japan) equipped with two grindstones (GC6-120, Masuko Sangyo, Japan) rotating at 1800 rpm. At this time, the distance between the grindstones was narrowed from the initial contact distance to 150 μm. This fibrillation treatment was repeated up to 4 times to obtain aqueous LCNF suspensions (Wet-DM-4).

Preparation of polymer composites

A composite material was prepared as previously reported procedure [S1]. A mixture of powdered PP (*i*PP homopolymer; NOVATEC MA3, Japan Polypropylene Co., Ltd.) and MAPP (Kayabrid 006PP, Kayaku Akzo Co., Ltd.) was used as the matrix. Wet LCNF (PP /LCNF = 90:5 w/w%) was subjected to solid-state shearing with PP powder at room temperature to 100 °C and rotation speed of 60 rpm using a Labo Plastomill R30 (Toyo Seiki Seisaku-sho, Ltd., Japan) for 30 minutes, followed by oven drying. Subsequently, it was combined with MAPP for melt mixing (PP with LCNF/MAPP = 95:5 w/w%) at 170°C and 30 rpm using a twin-screw extruder (4C150 Laboplastomill, 2D15W screw, Toyo Seiki Seisaku-sho, Japan) and cut into pellets. The pellets were molded using an injection molding machine (Babyplast 6/10P, Cronoplasto, S. L., Spain) at an extrusion molding temperature of 180°C and an injection molding temperature of 190°C. The injection pressure was 13 MPa and the mold temperature was the same as room temperature (about 25°C).

Izod impact test

The composite plates were stored in a room with a constant temperature of 22.5°C and a constant humidity of 30% for over 1 week, before the measurement. To minimize errors, each test group consisted of at least three specimens, and the average of the test results was calculated as the measured strength. The dimensions of the specimens tested were 3.8x9.6x58mm. The notched Izod impact strengths of the injection-molded composites were measured using a universal impact tester (No. 258-D; Yasuda Seiki Seisakusyo, Ltd., Japan). Notches were fabricated with a radius of 0.25 mm and a depth of 2 mm.

Table S1. Characteristics of 14 kinds of lignocellulosic fibrillated fillers with different mechanical preparation including dry pulverization (Dry-P) and subsequent wet disk milling for 4 and 10 passes (Wet-DM-4, Wet-DM-10, respectively).

Filler No.	Wood species	Preparation	SSA (m^2/g)	CI (%)	Sugar composition (%)				
					%Glc	%Xyl	%Gal	%Ara	%Man
F1	JC-I-M	Dry-P	5.45	40	49.6	5.6	0	0	3.1
F2		Wet-DM-4	17.3	39					
F3		Wet-DM-10	63.6	27					
F4	JC-I-J	Dry-P	4.65	38	47.7	6.0	0	0	11.5
F5		Wet-DM-4	22.2	34					
F6		Wet-DM-10	97.0	23					
F7	JC-T-M	Dry-P	5.06	38	43.2	4.7	2.5	1.3	6.3
F8		Wet-DM-4	19.9	40					
F9		Wet-DM-10	134	26					
F10	JC-T-J	Dry-P	4.01	37	42.8	5.6	3.5	1.9	7.7
F11		Wet-DM-4	25.2	26					
F12		Wet-DM-10	143	22					
F13	JC-K-M	Dry-P	5.33	36	40.2	4.6	2.8	1.3	8.3
F14		Wet-DM-4	20.4	36					
F15		Wet-DM-10	95.1	28					
F16	CF-J	Dry-P	4.62	37	46.9	4.9	3.3	1.4	9.1
F17		Wet-DM-4	30.4	36					
F18		Wet-DM-10	146	22					
F19	JL-M	Dry-P	5.12	39	40.6	4.1	4.7	1.7	10

F20		Wet-DM-4	16.3	36					
F21		Wet-DM-10	110	27					
F22		Dry-P	5.42	31					
F23	JL-J	Wet-DM-4	19.1	36	40.7	4.4	7.0	0.9	9.4
F24		Wet-DM-10	135	27					
F25		Dry-P	3.63	39					
F26	SF-M	Wet-DM-4	16.4	40	43.7	3.8	1.9	1.2	11.0
F27		Wet-DM-10	138	24					
F28		Dry-P	3.18	39					
F29	SF-J	Wet-DM-4	21.0	38	Na	Na	Na	Na	Na
F30		Wet-DM-10	113	27					
F31		Dry-P	5.14	27					
F32	WB-M	Wet-DM-4	13.4	31	45.2	21.9	0	0	2.4
F33		Wet-DM-10	51.8	26					
F34		Dry-P	3.95	33					
F35	WB-J	Wet-DM-4	13.8	31	33.5	17.1	0	0	1.7
F36		Wet-DM-10	31.9	30					
F37		Dry-P	4.45	37					
F38	EN-J	Wet-DM-4	12.7	36	43.7	9.6	0	0	1.7
F39		Wet-DM-10	54.7	31					
F40	MB	Dry-P	2.35	30	39.9	19.0	0	1.3	0
F41		Wet-DM-4	7.49	29					

F42	Wet-DM-10	35.2	26

Abbreviations of wood fillers: **Wood kind-(provenance)-Age.**

Wood types: JC, Japanese cedar; CF, Chinese fir; JL, Japanese larch; SF, Sakhalin fir; WB, White birch; EN, Eucalyptus nitens; MB, Moso bamboo.

Provenance: I, Ibaraki Prefecture; T, Toyama Prefecture; K, Kumamoto Prefecture.

Age: J, Juvenile; M, Mature.

SSA: specific surface area; CI: crystallinity index.

Table S2. Tensile and Izod impact properties of PP/nanofibrilled cellulose composites.

Comp. No.	Sample	Process	Tensile strength (MPa)	Tensile breaking strain (%)	Young's modulus (MPa)	Flexural modulus (MPa)	Ultimate strength (MPa)	Izod impact energy (kJ/m^2)	Training dataset	Test dataset
	PP	-	38.8±1.3	960±108	815±72	1810±24	58.6±0.3	1.05±0.022		
	PP+MA PP	-	37.8±1.2	688±62	1191±24	1792±30	57.6±0.3	1.22±0.024		
C1	JC-I-M	Dry-P	36.6±0.8	102±5	952±140	2149±23	64.8±0.4	1.21±0.11		✓
C2		Wet-DM-4	37.1±0.5	63±34	1094±40	2154±10	64.6±0.3	1.34±0.28	✓	
C3		Wet-DM-10	36.9±0.6	472±44	1142±25	2081±25	63.5±0.6	1.12±0.12		✓
C4	JC-I-J	Dry-P	36.6±0.4	74.0±11	1126±62	2104±52	65.2±0.5	1.08±0.068	✓	
C5		Wet-DM-4	36.8±0.6	126±34	1112±53	2132±10	64.7±0.4	1.12±0.12	✓	
C6		Wet-DM-10	36.4±0.2	462±45	1086±30	2094±48	64.4±0.4	0.95±0.081		✓
C7	JC-T-M	Dry-P	37.0±0.3	65±32	1114±25	2054±39	62.9±0.4	1.45±0.12	✓	

C8		Wet-DM-4	36.6±0.2	85±15	1100±24	2091±15	63.7±0.2	1.06±0.071	✓	
C9		Wet-DM-10	36.8±0.4	445±21	1106±21	2070±19	63.3±0.3	1.13±0.15		✓
C10		Dry-P	37.1±0.3	46±14	1110±53	2176±26	64.7±0.4	1.71±0.30	✓	
C11	JC-T-J	Wet-DM-4	36.4±0.4	102±28	1084±16	2117±44	64.2±0.6	1.48±0.33	✓	
C12		Wet-DM-10	36.4±0.3	502±14	1076±27	2067±56	63.0±1.3	1.23±0.088	✓	
C13		Dry-P	37.8±0.2	102±18	1057±70	2175±20	65.2±0.3	1.50±0.37	✓	
C14	JC-K-M	Wet-DM-4	37.3±0.2	76±22	1038±51	2172±13	65.0±0.3	1.69±0.35		✓
C15		Wet-DM-10	37.8±0.2	159±129	935±106	2123±22	64.1±0.3	1.40±0.077	✓	
C16		Dry-P	37.3±0.1	28±17	1041±36	2142±21	64.9±0.2	1.83±0.33	✓	
C17	CF-J	Wet-DM-4	36.7±0.3	101±12	1064±41	2090±11	63.2±0.16	1.63±0.39		✓
C18		Wet-DM-10	35.7±0.1	558±53	1015±22	2053±25	62.1±1.1	1.34±0.11		✓

C19		Dry-P	37.4±0.1	95±12	1054±63	2077±17	63.4±0.1	1.70±0.23		✓
C20	JL-M	Wet-DM-4	37.1±0.3	77±13	1035±71	2034±57	62.7±1.3	1.48±0.11	✓	
C21		Wet-DM-10	36.8±0.1	444±6	988±62	2018±36	62.9±0.9	1.37±0.087	✓	
C22		Dry-P	37.1±0.2	115±25	993±44	2101±46	64.2±1.5	1.71±0.40	✓	
C23	JL-J	Wet-DM-4	37.1±0.3	107±12	1020±33	2070±40	64.1±1.4	1.62±0.39	✓	
C24		Wet-DM-10	37.1±0.2	451±19	1031±68	2013±25	62.8±0.8	1.37±0.16	✓	
C25		Dry-P	37.3±0.7	95±12	1020±53	2166±22	65.4±0.2	1.50±0.20	✓	
C26	SF-M	Wet-DM-4	36.2±0.4	77±13	1011±20	2181±11	66.0±0.5	1.34±0.067	✓	
C27		Wet-DM-10	36.1±0.2	443±6	1005±57	1970±47	62.0±0.7	1.22±0.19	✓	
C28	SF-J	Dry-P	37.5±0.3	78±8	992±44	2168±20	65.4±0.4	1.45±0.36	✓	
C29		Wet-DM-4	37.2±0.3	106±12	1073±18	2031±39	62.8±0.8	1.31±0.098	✓	

C30		Wet-DM-10	36.9±0.4	452±19	1030±14	2007±15	62.4±0.2	1.33±0.15	√	
C31	WB-M	Dry-P	38.6±0.5	61±3	1031±15	2093±29	63.7±0.6	2.10±0.25	√	
C32		Wet-DM-4	38.2±0.2	84±20	1055±46	2068±51	63.3±1.1	2.09±0.37	√	
C33		Wet-DM-10	37.6±0.2	255±144	1071±31	2078±23	63.5±0.5	1.74±0.19	√	
C34	WB-J	Dry-P	38.1±0.2	70±7	1076±32	2140±44	64.9±1.5	2.09±0.26		√
C35		Wet-DM-4	38.3±0.1	59±34	1058±38	2063±8.0	63.0±0.1	2.07±0.30	√	
C36		Wet-DM-10	38.1±0.7	69±7	1062±40	2061±43	63.1±1.3	1.87±0.20	√	
C37	EN-J	Dry-P	36.2±0.5	70±58	1113±27	2059±12	62.8±0.3	1.96±0.23		√
C38		Wet-DM-4	36.2±0.3	50±5	1089±28	2039±28	62.6±0.9	1.89±0.35		√
C39		Wet-DM-10	34.9±0.3	434±81	1015±80	1987±20	61.5±0.4	1.77±0.36	√	
C40	MB	Dry-P	36.0±0.2	23±16	1132±25	2065±6.8	62.6±0.2	2.04±0.25		√

C41		Wet-DM-4	35.8±0.1	21±10	1113±18	2055±11	62.5±0.2	1.89±0.35		✓
C42		Wet-DM-10	35.7±0.1	101±14	1027±59	2003±16	62.2±0.3	2.16±0.12	✓	

Table S3. Wood species for discovering promising composite materials with high izod impact energy.

	Name	Tree Species
S1	Agathis	Softwood
S2	Balsam	
S3	Ginkgo	
S4	Japanese cypress	
S5	Japanese fir	
S6	Lawson cypress	
S7	Radiata pine	
S8	Red pine	
S9	Spruce	
S10	Western hemlock	
S11	Yellow cedar	
S12	Yellow pine	
S13	Yoshino cedar	
S14	Apitong	Hardwood
S15	Ash	
S16	Basswood	
S17	Beech	
S18	Camphor	
S19	Castor aralia	
S20	Cherry	
S21	Elm	
S22	Hard maple	
S23	Japanese ash	
S24	Japanese chestnut	
S25	Japanese horse chestnut	
S26	Japanese linden	
S27	Japanese white birch	
S28	Magnolia	
S29	Maple	
S30	Monarch bbirch	
S31	Nyatoh	
S32	Oak	
S33	Paulownia	

S34	Persimmon
S35	Red cedar
S36	Red oak
S37	Red-bark oak
S38	Teak
S39	Walnut
S40	White ash
S41	White oak
S42	Wild cherry
S43	Yellow birch
S44	Yellow poplar
S45	Zelkova

Table S4. Assignment of ATR-FT-IR bands for lignocellulosic fillers.

Measured region	Ref. cellulose (cm^{-1})	Ref. lignin (cm^{-1})	Ref. xylan-derived unit (cm^{-1})	Ref. mannan-derived unit (cm^{-1})	Assignment	Supporting References
1		1737	1734-1730	1734-1730	ν (C=O) glucuronic acid (GlcA) unit	[S2-5]
2		1660-1655			ν (C=O)	
	1640-1636		1640-1630	1640	ν(H-O-H) moisture adsorption	
3		1603-1596			δ (C=C) Arom. ring	[S2-4,7,8]
4		1557-1505			δ (C=C) Arom. ring	[S2-5,7,8]
5	1456-1454	1464-1462	1474-1454	1454	δ(CH_2), δ(CH_3)	[S2,4-8]
6	1433-1415	1427-1422	1422-1408	1422-1417	δ scissoring (CH_2) (CH_3) of lignin	
7	1375-1370	1385–1370	1386-1370	1380-1370	δ (CH) – δ_s (CH_3)	[S2-4,6,7]
8	1355-1314	1352–1326	1355-1313	1355-1309	γ (CH_2), δ (O-H) δ (CH_2)	[S2,4,6-8]
9	1268-1238	1269-1215	1252-1220	1260	ν (C-O)	[S2,3,6-8]

10	1205		1208-1205	1208-1205	δ (CH_2)<br>δ (O—H)	
11	1165-1156	1146-1134	1165-1156	1180-1156	ν_{as}(C—O—C)	[S2,4-8]
12	1112-1108		1115-1110	1141-1108	ν (C—O)<br>ν (O—H)	
	1083-1052	1080	1082-1052	1125-1052	ν (C—O)	
13	1056-1028	1032-1130	1045-1030	1130	ν_s (C—O—C)	
14	898-895		898	898-871	ν (C—C)	
	850	860-853		870	δ (C—H)	
	811	817-812		810-710	δ (C—C);	[S4-6, 8-10]

ν: stretching, δ: bending,

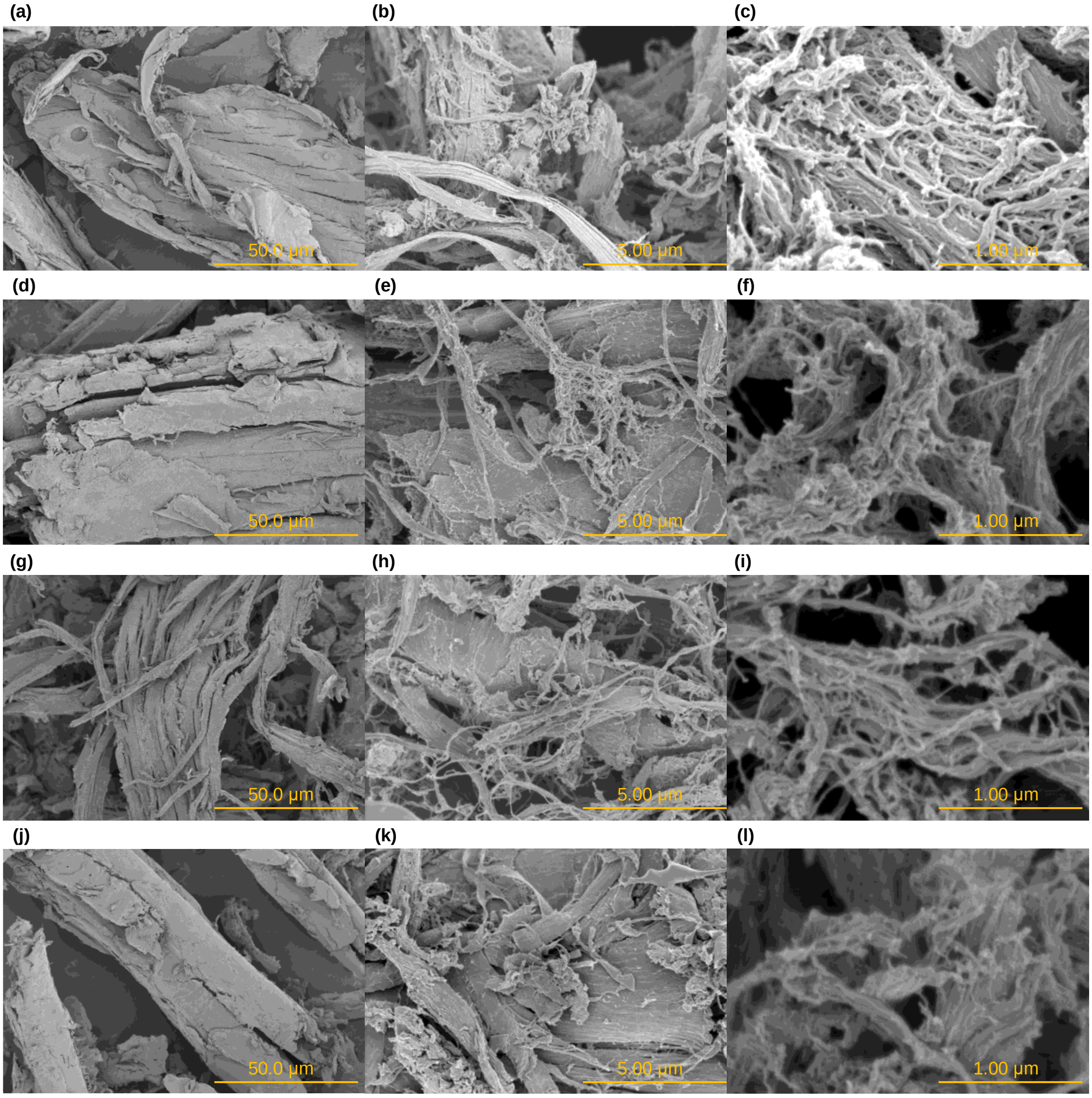

Figure S1. a-c) JC-I-M, d-f) JL-M, g-i) EN-J, j-l) MB with each process for Dry-P, JL-M W-DM-4, respectively.

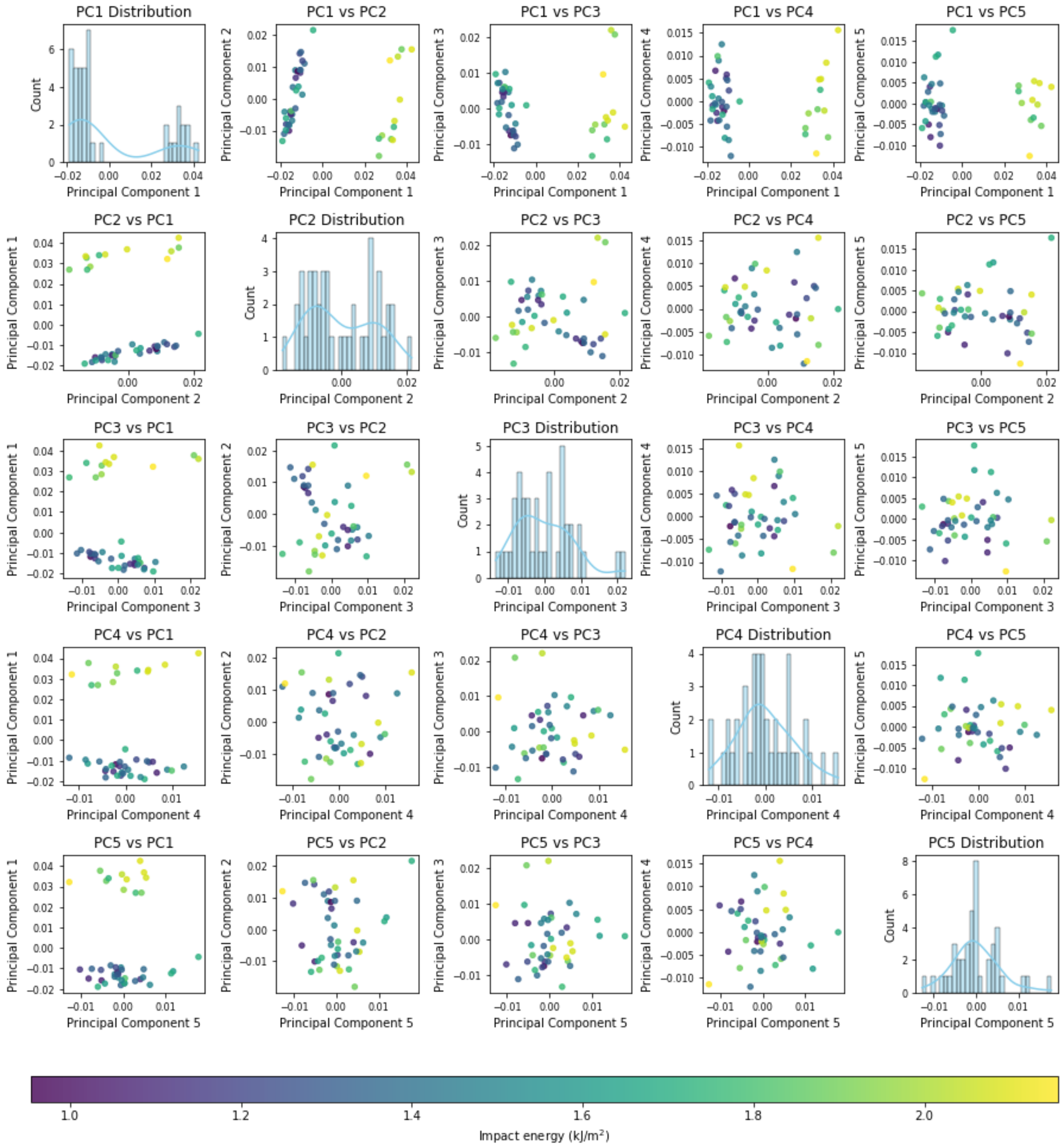

Figure S2. Loading plot representing the contribution of the original variables for the principal components PC1 to PC5. As variables that contribute most are plotted around the borders of the plot, the inset shows a typical ATR-IR spectra, which was set up with smoothing and 2nd deviation.

Supporting References

S 1. Iwamoto S, Yamamoto S, Lee S-H, et al. Solid-state shear pulverization as effective treatment for dispersing lignocellulose nanofibers in polypropylene composites. Cellulose. 2014;21(3):1573-1580.

S 2. Popescu C-M, Popescu M-C, Vasile C. Characterization of fungal degraded lime wood by FT-IR and 2D IR correlation spectroscopy. Microchem J. 2010;95(2):377-387.

S 3. Cheng SC, Huang AM, Wang SN, et al. Effect of Different Heat Treatment Temperatures on the Chemical Composition and Structure of Chinese Fir Wood. Bioresources. 2016;11(2):4006-4016.

S 4. Horikawa Y, Hirano S, Mihashi A, et al. Prediction of Lignin Contents from Infrared Spectroscopy: Chemical Digestion and Lignin/Biomass Ratios of Cryptomeria japonica. Appl Biochem Biotechnol. 2019;188(4):1066-1076.

S 5. Liu X, Renard C, Bureau S, et al. Revisiting the contribution of ATR-FTIR spectroscopy to characterize plant cell wall polysaccharides. Carbohydr Polym. 2021 Jun 15;262:117935.

S 6. Xiong J, Li Q, Shi Z, et al. Interactions between wheat starch and cellulose derivatives in short-term retrogradation: Rheology and FTIR study. Food Res Int. 2017;100(Pt 1):858-863.

S 7. Pandey KK, Pitman AJ. FTIR studies of the changes in wood chemistry following decay by brown-rot and white-rot fungi. Int biodeterior biodegrad. 2003;52(3):151-160.

S 8. Boeriu CG, Bravo D, Gosselink RJA, et al. Characterisation of structure-dependent functional properties of lignin with infrared spectroscopy. Ind Crops Prod .. 2004;20(2):205-218.

S 9. Barsberg S, Sanadi AR, Jørgensen H. A new Density Functional Theory (DFT) based method for supporting the assignment of vibrational signatures of mannan and cellulose—Analysis of palm kernel cake hydrolysis by ATR-FT-IR spectroscopy as a case study. Carbohydr Polym. 2011;85(2):457-464.

S 10. Kato K, Nitta M, Mizuno T. Infrared Spectroscopy of Some Mannans. Agric Biol Chem. 1973;37(2):433-435.
